# Sphingosine-1-Phosphate Metabolic Pathway in Cancer: Implications for Therapeutic Targets

**DOI:** 10.3390/ijms26031056

**Published:** 2025-01-26

**Authors:** Miguel L. Rufail, Rosaria Bassi, Paola Giussani

**Affiliations:** 1 Department of Pathology, Virginia Commonwealth University, Richmond, VA 23298, USA; 2 Department of Medical Biotechnology and Translational Medicine, Università degli Studi di Milano, LITA Segrate, Via Fratelli Cervi, 93, 20054 Segrate, Italy

**Keywords:** sphingolipids, sphingosine-1-phosphate (S1P), cell growth, drug resistance, cancer

## Abstract

Cancer biology revolves around understanding how cells undergo uncontrolled proliferation leading to the formation of malignant tumors. Key aspects include self-sufficiency in growth signals, the lack of response to signals of growth inhibition, the evasion of apoptosis, sustained angiogenesis, the evasion of immune response, the capacity to invade and metastasize, and alterations in cellular metabolism. A vast amount of research, which is exponentially growing, over the past few decades highlights the role of sphingolipids in cancer. They act not only as structural membrane components but also as bioactive molecules that regulate cell fate in different physio-pathological conditions. In cancer, sphingolipid metabolism is dysregulated, contributing to tumor progression, metastasis, and drug resistance. In this review, we outline the impact of sphingosine-1-phosphate (S1P) as a key bioactive sphingolipid in cancer. We give an overview of its metabolism summarizing the role of S1P as an intracellular and extracellular mediator through specific plasma membrane receptors in different cancers. We also describe previous findings on how the disruption in the balance between S1P and ceramide (Cer) is common in cancer cells and can contribute to tumorigenesis and resistance to chemotherapy. We finally consider the potential of targeting the metabolic pathways of S1P as well as its receptors and transporters as a promising therapeutic approach in cancer treatments.

## 1. Introduction

Cancer is the second most common cause of death after cardiovascular disease. In the past few decades, there has been an immense advancement in our understanding of its pathogenesis and biology. In this disease, there is an uncontrolled proliferation of cells which invade and destroy adjacent structures and spread to distant sites in a process called metastasis. There are multiple factors that drive, perpetuate and exacerbate the pathogenesis of cancer, including inherited predisposition, environmental interactions and advanced age. The key aspects of cell biology that are responsible in the pathogenesis of cancer are as follows: self-sufficiency in growth signals; the lack of response to signals of growth inhibition; alterations in cellular metabolism; the evasion of apoptosis; unrestricted proliferative capacity; sustained angiogenesis; the evasion of immune response; and the capacity to invade and metastasize [1].

Many mechanisms at the biochemical and molecular levels have been implicated in the aforementioned cellular processes that define cancer. In the past few decades, bioactive lipids with their associated metabolism and regulatory pathways have emerged as important players in the pathogenesis and potential treatment of cancer. Lipids, and, in particular, sphingolipids, as bioactive molecules have crucial roles in the control and regulation of cell fate in different physiological and pathophysiological conditions [2,3]. Sphingolipid metabolism has the role to regulate the levels of the different bioactive lipids. The dysregulation of lipids is a hallmark of different pathological conditions including tumor progression [4,5,6,7,8]. Several lipids have been identified as prognostic markers [9], among them a variety of sphingolipids such as sphingosine-1-phosphate (S1P), ceramide (Cer), dihydroceramide, glucosylceramide (GlcCer), and lactosylceramide. These molecules as well as the enzymes involved in their metabolism can be considered potential therapeutic targets.

Cer is the central molecule of sphingolipid metabolism and can be synthesized via two major pathways: “de novo synthesis” and the salvage pathway that utilizes the sphingolipid breakdown product sphingosine, which can be reacylated with fatty acid to form Cer.

Cer can be degraded to sphingosine (Sph), which, in turn, can be phosphorylated to generate S1P. Spiegel and her collaborators highlighted the crucial role of the sphingolipid (S1P/Cer) rheostat in the regulation of cell fate. In particular, the balance between S1P and Cer levels is crucial in the initiation, progression, and drug sensitivity of cancer [6,10]. S1P and Cer regulate opposite processes; S1P promotes proliferation, angiogenesis, metastasis, survival and drug resistance [6,11,12,13,14]. Cer is instead able to induce cell growth arrest and cell death [6]. On these bases, regulation of the S1P/Cer rheostat has emerged as a target for treatment strategies to treat cancer. In this review, we will focus on the bioactive sphingolipid S1P, highlighting (1) the crucial role that it has in cancer, (2) the implications of its alterations in cancer, and (3) its potential as target in cancer therapies.

## 2. Sphingolipid Metabolism

Sphingolipids are essential components of all eukaryotic cells and play important roles in cell membrane structure and function. Beyond they structural roles, some of them, like Cer and its metabolites, along with S1P, have emerged as bioactive molecules involved in the regulation of numerous cellular processes [8,11,15]. They can originate from “de novo synthesis” to form Cer (Sph linked to a fatty acid). The “de novo” synthesis begins with the condensation of palmitoyl-CoA with L-serine, catalyzed by serine palmitoyl transferase, to form dihydrosphingosine (sphinganine). Subsequently, sphinganine binds to a fatty acid, forming dihydroceramide in a reaction promoted by a family of six (dihydro)-ceramide synthases (CerS). The last step of “de novo” synthesis is the addition of a double bond by dihydroceramide desaturase to form Cer. Cer can derive also from the salvage pathway in which CerS catalyzes the synthesis from Sph [8,15]. Cer can be phosphorylated by Cer kinase to form Cer 1-phosphate (Cer1P) [8]. Cer, in turn, can be converted to complex sphingolipids (sphingomyelin (SM) and glycosphingolipids). SM is generated when the 1-hydroxyl group of Cer is linked to phosphocholine in a reaction catalyzed by SM synthase (SMS existing in two isoforms) [8]. Glycosphingolipids are generated when the 1-hydroxyl group of Cer is linked to one or more saccharide units, mediated by Cer galactosyl transferase or glucosylceramide synthase to form galactosylceramide or GlcCer, respectively [8,15]. GlcCer can be further glycosylated giving rise to more complex sphingolipids such as gangliosides [8,15]. SM can be degraded by sphingomyelinase (SMase), existing in three different groups according to their localization and optimum pH, to form Cer and phosphocholine. Cer can be de-acylated by three isoforms of ceramidases (classified according to their optimum pH), giving rise to Sph and fatty acids [8,15]. Sph, in turn, is phosphorylated by sphingosine kinases to form S1P.

**Figure 1:**
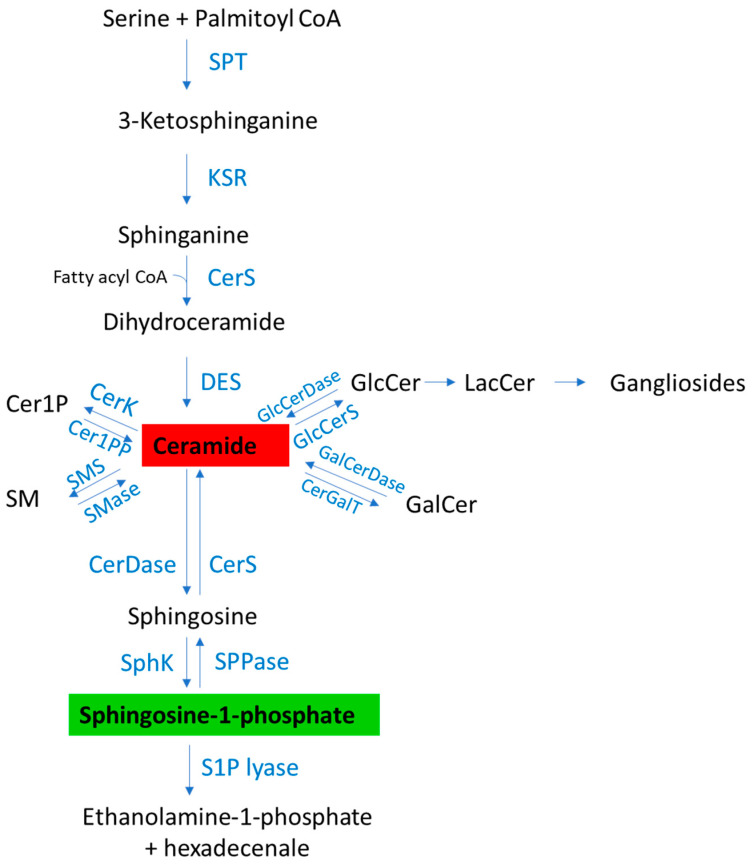
Sphingolipid synthesis and metabolism. SphK, sphingosine kinase; SPPase, S1P phosphatase; SPT, serine-palmitoyl transferase; KSR, ketosphinganine reductase; CerS, ceramide synthase; DES, dihydroceramide desaturase; GlcCerS, glucosylceramide synthase; GlcCerDase, glucosylceramidase; CerGalT, ceramide galactosyl transferase; GalCerDase, galactosylceramidase; Cer1PP, ceramide-1-phosphate phosphatase; CerK, ceramide kinase; SMS, sphingomyelin synthase; SMase, sphingomyelinase; SPP, S1P phosphatase; Cer, ceramide; SM, Sphingomyelin; Cer1P, Ceramide-1-phosphate; GlcCer, Glucosylceramide; GalCer, Galactosylceramide; LacCer, Lactosylceramide.

Two isoforms of sphingosine kinase are known: sphingosine kinase 1 (SphK1) found predominantly in the cytosol [16], and sphingosine kinase 2 (SphK2) with a localization mainly at the plasma membrane and nucleus [17,18]. S1P can be irreversibly cleaved to hexadecenal and phosphoethanolamine by S1P lyase, localized at the ER [19,20,21], or can be dephosphorylated to Sph by specific S1P phosphatases (SPPs) [22,23,24,25,26]. S1P levels are tightly regulated by the finely tuned balance between synthesis and degradation. An overview of the sphingolipid biosynthetic and catabolic pathways is shown in Figure 1.

## 3. S1P Functions

**Figure 2:**
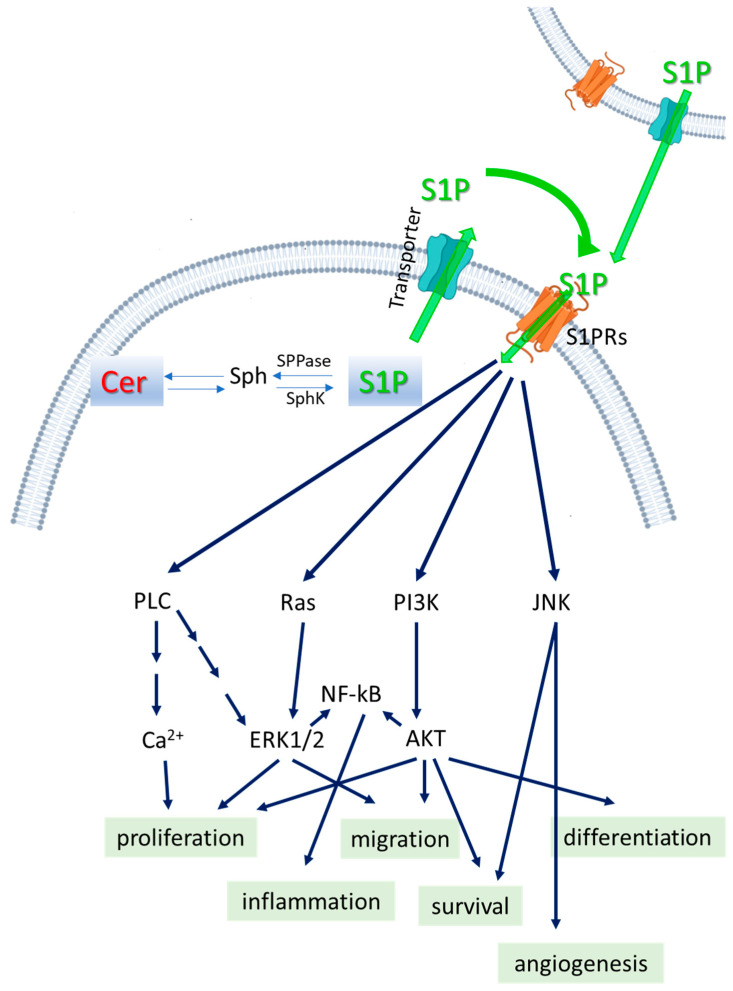
S1P signaling pathways. Schematic cartoon of the enzymes that regulate the S1P levels with “inside-out” signaling by the S1P/S1PR axis and the main downstream signaling pathways, such as PLC, AKT, ERK, and JNK, leading to the modulation of cell proliferation, migration, differentiation, survival, angiogenesis and inflammation. SphK, sphingosine kinase; SPPase, S1P phosphatase; S1PRs, S1P receptors.

S1P is a bioactive molecule involved in the regulation of a large number of cellular processes, including the physiology and physiopathology of the central nervous system, cell migration and invasion, cell cycle, inflammation, immune system modulation, vascular function, angiogenesis, and cancer [11,14,27,28]. Its multi-faceted role in the biology of cancer has been widely recognized, as it can influence tumor growth, resistance to apoptosis, metastasis, angiogenesis, immune evasion and therapy resistance [13,15,29,30,31,32]. There is ample evidence on the role of S1P in specific different cancer types, such as breast, ovarian, gastrointestinal, hepatocellular carcinoma, glioblastoma multiforme, bladder, pancreatic cancer, and colorectal and hematological malignancies, even if its effects are different depending on the tumor type, stage, and the specific receptor subtypes involved. Indeed, many of the effects of S1P are exerted through its binding to a family of G-protein-coupled receptors, which mediate S1P signaling pathways [13,14,33,34,35,36,37,38,39], activating various signaling pathways such as PI3K/Akt, MAPK and NF-kB, as illustrated in Figure 2.

Recent research has shown that S1P and its signaling pathways can influence epigenetic regulation in cancer, affecting DNA methylation, histone modification and non-coding RNA regulation [40,41,42,43]. For example, S1P and its analog FTY720-P can affect histone acetyltransferases and histone deacetylases, which in turn influence chromatin accessibility and gene expression. In some cancers, altered S1P signaling leads to the activation of pro-survival genes through epigenetic regulation of histones enabling growth and metastasis. In addition, there is growing evidence that S1P can also influence the expression of non-coding RNAs, which in turn modulate the expression of genes involved in tumor progression. Moreover, S1P signaling contributes to epithelial-to-mesenchymal transition, a key process in metastasis that is also regulated by epigenetic mechanisms. This suggests a possible interplay between these processes that may create a favorable environment for metastasis. The findings mentioned above, indicate that S1P has a multifaceted role in cancer epigenetic alterations and further studies are needed to better understand how these pathways interact to provide a potential therapeutic strategy to target S1P signaling in cancer and overcoming metastasis and chemotherapy resistance.

## 4. Targeting S1P Metabolic Enzymes to Modulate the Sphingolipid Rheostat for Cancer Therapy

Targeting the enzymes involved in sphingolipid metabolism has emerged as an area of great interest in drug development due to their involvement in various processes fundamental in cancer. As we mentioned above, the balance between S1P and Cer levels is crucial in the initiation, progression, and drug sensitivity of cancer. On these bases, we will focus our review on the effect of targeting the key enzymes involved in S1P metabolism [6,44,45,46].

### 4.1. SphK1

S1P antagonizes Cer-mediated apoptosis through ERK activation and Cer-induced JNK activation [10,47]. SphK1, localized mainly in the inner layer of the plasma membrane, generates S1P after translocation to the plasma membrane [48]. It has been shown that many cancers are characterized by high levels of SphK1 and this is directly correlated with poor prognosis, reduced survival, and advanced tumor stage [6,29,49,50]. Several lines of evidence link the oncogenic role of S1P to the activity of SphK1: (i) SphK 1 and S1P levels regulate drug and radiation resistance in cell cultures; (ii) overexpression of SphK1 and an increase in S1P levels drive tumors toward more aggressive forms in animal models, while SphK1 inhibition restores drug and radiation sensitivity; (iii) SphK1 is overexpressed in many types of tumor, and it has been shown that high levels of SphK1 directly correlate with poor survival in patients. Crucial roles for S1P/SphK1 in promoting cell survival, angiogenesis, migration, and drug resistance have been shown [13,14,29,51]. In particular, S1P/SphK1 is intrinsically involved in drug resistance; this signaling pathway protects cancer cells from chemotherapy-induced apoptosis. For example, Bektas et al. demonstrated that in melanoma cells, the overexpression of SphK1 makes the cells less sensitive to Cer-mediated apoptosis, but the downregulation of SphK1 expression reverses this effect [52]. Moreover, in breast cancer cells, SphK1 overexpression promotes cell proliferation and resistance to tamoxifen, but the downregulation of the enzyme restores tamoxifen’s effect [53]. Furthermore, in prostate adenocarcinoma, SphK1 is involved in the regulation of drug-induced apoptosis in cell cultures as well as in animal models [54]. Recent studies support the relevant contribution of SphK activity to drug resistance in cancer cells by regulating key apoptosis pathways. Elevated SphK activity promotes survival signaling and inhibits apoptosis induced by cisplatin and doxorubicin [55,56].

Interestingly, human glioblastoma EGF receptor-overexpressing cells, characterized by a high level of extracellular S1P and increased SphK1 activity, are resistant to temozolomide, and the inhibition of SphK1 or S1P receptors made the cells sensitive to the drug, demonstrating a functional link between S1P and EGFR signaling pathways [13]. The SphK/S1P pathway protects cancer cells from drug-induced cell death, demonstrating that the regulation of S1P levels is involved in the modulation of drug resistance [57,58,59]. In another study, the crosstalk of S1P and receptor tyrosine kinase-like orphan receptor ROR1/2 signaling pathways, which are activated in lung carcinogenesis cells, was evaluated. S1P treatment decreased ROR1/2 transcript levels, while the opposite effect occurred when a SphK1 inhibitor was used, pointing to reciprocal regulation and potential simultaneous therapeutic targeting of both pathways [60]. Using Kaplan–Meier plotter, an online database containing the gene expression information of around 2000 lung cancer patients, Wang et al. [61] found that high SPHK1 mRNA expression was significantly correlated with worse overall survival, while high SPHK2 mRNA expression was in favor of better overall survival, in keeping with some of the findings of other studies mentioned above. SphK1 inhibitors are considered potential drugs [62] since SphK1 is often upregulated in cancer and associated with different processes of tumorigenesis. SphK1 inhibition sensitizes cancer cells to chemotherapeutic agents.

Several inhibitors have been designed for SphK1; French et al. evaluated a large number of non-lipid synthetic inhibitors of SphK1 and demonstrated that they induced apoptosis and cell cytotoxicity in many cancer cell lines, as well as in multidrug-resistant cell lines [63]. Moreover, the sphingolipid analog N,N-dimethylsphingosine (DMS) and SK1-II, that have poor potency and selectivity between SphK isoforms, and SK1-I, specific for SphK1, demonstrated promising results [64]. DMS inhibits cell growth and reduces the in vivo metastasis of leukemia, colon, epidermoid, and lung tumors [65,66], and increases the sensitivity to apoptosis induced by radiation of human leukemia cells [67]. The “second generation” of SphK inhibitors derive from an intensive screening program of many pharmaceutical companies. The most promising compound, PF-543, is considered the most potent SphK1-selective inhibitor. It has shown a promising profile for sickle cell disease [68]. Further results demonstrate that PF-543 inhibits the ability of extracellular S1P to promote human glioblastoma cell survival and invasiveness [13,14]. On the other hand, different authors demonstrate SphK1 inhibition by PF-543, showing that it had no effect on the proliferation and survival of cancer cells, even if S1P levels were modified [69]. Recently, many new molecules such as SKI-178, B-5335c, and compound 51/82 were designed to specifically inhibit SphK1, but all these inhibitors need to be validated in tumor growth and metastasis models [70]. It has been shown that the decreased expression of SphK1 improves the efficacy of immune checkpoint inhibitors such as anti-CTLA-4 and anti-PD-1 therapy in melanoma, breast, and colon cancer mouse models [71,72]. Further studies are necessary to evaluate the efficacy and the effects of SphK1 inhibitors in the regulation of cell fate.

### 4.2. SphK2

In contrast to SphK1, SphK2’s role in cancer is not fully understood [6,73]. SphK2 is localized in several subcellular compartments, particularly in the nucleus, and there is significant debate over controversial results regarding the role of this enzyme in carcinogenesis. Several studies demonstrated that SphK2 overexpression promotes apoptosis and inhibits cell growth [17,74,75,76]. In particular, Liu et al. demonstrated that SphK2 can be a pro-apoptotic molecule [76]. It has been shown that SphK2 expression sensitizes cells to chemotherapeutic agents [77]. Recently, a crucial role in the cancer progression of S1P elicited by SphK2 has been shown. The selective SphK2 inhibitor ABC294640 decreases cancer cell growth in vitro and in mouse models of cancer [78,79,80], even though this inhibitor has also been linked to off-target anti-estrogenic effects [81]. Moreover, a different compound, SLR080811, reduced the level of S1P in cells but did not reduce cell proliferation, while it induced an increase in blood S1P levels in mice [82]. In addition, inhibition of SphK2 precluded the effect of S1P in lung cancer cell proliferation [83], which has been linked to TNF-α release through the induction of Toll-like receptor 9 [84]. Recently, it has been demonstrated that high levels of SphK2 are directly associated with aggressive hepatocellular carcinoma differentiation and more frequent cancer recurrence in males [85]. Additional data show that the inhibition or knockdown of SphK1 and SphK2 (i) decrease glioblastoma cell proliferation [50,86] and (ii) make glioma stem cells sensitive to temozolomide [87,88]. Contrary to SphK1, which generates S1P and decreases Cer formation, SphK2 plays a role in the salvage pathway, acting together with S1P phosphatase (SPP1) to convert S1P to Sph, which is subsequently converted to Cer [89,90]. In addition, FTY720, an Sph analog that is also known as fingolimod, is an FDA-approved drug used to treat multiple sclerosis; it works by modulating S1P receptors after its activation is promoted by SphK2 phosphorylation [91]. All together, these data demonstrate a crucial relationship between the modulation of S1P metabolism and drug resistance in human cancer cells while indicating that further studies are needed to target SphK2 in the treatment of cancer.

### 4.3. S1P Lyase and S1P Phosphatases

S1P lyase, which regulates S1P levels by enacting the irreversible catabolism of S1P, has been shown to also regulate Cer and Sph levels. The knockdown of S1P lyase in mice is characterized by altered S1P signaling and inflammatory responses, leading to early death [92,93]. The levels of S1P lyase are downregulated in different human cancers and there is an inverse correlation with clinical outcomes and resistance to treatment [6,93]. It is known that intestinal S1P lyase deletion, through S1P/S1PR1, promotes colon carcinogenesis, activating STAT3 [94]. In fibroblasts deficient in S1P lyase, there are high levels of S1P in the nucleus with reduced histone deacetylase activity, which causes the dysregulation of Ca^2+^ homeostasis and upregulation of S1P transporters. Among them, ABC transporters give rise to chemoresistance [95,96].

There are two different S1P phosphatases (SPP1 and SPP2) which participate in the regulation of S1P levels. It is known that the expression of SPPs is decreased in many types of cancer [6]. In particular, increased S1P levels in tumors are directly correlated with high levels of SphK1 and low levels of SPP2, further suggesting complex coordination in the regulation of sphingolipid metabolism [97].

### 4.4. S1P and Its Receptors

S1P generated by SphKs can act both as an intracellular as well as an extracellular mediator. S1P can be secreted and act in an autocrine or paracrine manner [98,99] through G-protein-coupled receptors specific for S1P (S1PR1-5) [100,101]. These receptors are coupled to different signaling pathways, such as Akt/mTOR, NF-κB, and MAPK (Figure 2) [74,102,103,104]. The signaling axis of S1P-S1PRs promotes cell growth, survival, motility, and angiogenesis, all critical roles in the pro-oncogenic S1P effect [29,51,105]. S1P binding to several S1PRs activates the ERK and/or AKT signaling pathways downstream to promote survival [6], while S1P binding to S1PR3 promotes the activation of the mTOR pathway that counteracts ceramide-mediated autophagy [106]. In lung adenocarcinoma cells, S1PR3 is increased and elicits EGFR expression. In addition, S1P treatment promotes the proliferation and invasion of lung adenocarcinoma cells [107]. S1PR3 was found to be more abundant in human lung adenocarcinoma tissue compared to normal tissue and was linked to S1P-mediated proliferation of adenocarcinoma cells [83]. Liang et al. demonstrated that the S1P/S1PR1 axis links the persistent activation of STAT3, chronic intestinal inflammation, and the development of colitis-associated cancer [108]. In general, S1P binds to S1PRs and in turn activates different downstream signaling pathways through G proteins [109]. In particular, S1PR1 and S1PR4 bind to Gi; S1PR2 to Gi, G12/13, and Gq; S1PR3 to Gi, G12/13, and Gq; and S1PR5 to Gi and G12 [109,110,111,112,113]. It has been demonstrated that in numerous cancers, the S1P/S1PR1 axis activates different signaling pathways: JAK/STAT3, AKT, and PI3K [114,115]. On the other hand, the S1P/S1PR2/3 and S1PR4 axes, when coupled to Gi, activate different signaling pathways: ERK, PI3K, Yes-associated protein, and AKT [114,115,116,117]. The S1P/S1PR5 axis activates the focal adhesion kinase (FAK) signaling pathways [118]. Another strategy to modulate S1P signaling could be the regulation of the S1PRs using specific inhibitors such as FTY720-P, SEW2871, JTE 013, and VPC 23019 [119]. Recently, Singh et al. demonstrated that when used in breast tumor-bearing immunocompetent mice, FTY720 potentiates the effects of paclitaxel, significantly reducing tumor progression and lung metastasis [120]. A list of clinical trials targeting S1P pathways in different types of cancer, based on the currently available clinical data, is shown in Table 1.

### 4.5. S1P Transporters

For several years, it was not known how S1P produced intracellularly by SphKs was able to exit cells to act through S1PRs, but now, it has been demonstrated that S1P is exported to the extracellular milieu through Spinster 2 (Spns2) (according to the electrochemical gradient without consuming ATP) [121,122,123], as well as through ABC transporters [6,99,124,125,126]. Spns2 is present in different organelles, cells and tissues depending on the species. Spns2 is highly expressed in the liver and lung in mice [123,127]. On the other hand, Spns2 is expressed in most human tissues. It is abundant in the lung, placenta and stomach, but moderate levels are found in the brain, small intestine, uterus, skin, and lymph nodes, and very low levels are found in the hematopoietic tissues and blood vessels [123,128]. For example, the S1P transporter Spns2 has been shown to regulate cell migration and apoptosis in lung adenocarcinoma cells [129]. S1P secreted by the transporter from tumor cells can induce the proliferation and invasiveness of tumors but can also improve angiogenesis [130] and influence tumor immunity [131,132]. In addition, van der Weyden et al. demonstrated that Spns2-deficient mice were characterized by the largest reduction in pulmonary metastasis [133]. Moreover, they demonstrated that the deletion of Spns2, either globally or in a lymphatic endothelial-specific manner, creates a circulating lymphopenia and a higher percentage of effector T cells and natural killer (NK) cells present in the lung [133]. Therefore, the inhibition of the S1P transporter Spns2 could be another strategy to modulate S1P signaling, but specific inhibitors targeting Spns2 are not currently available.

### 4.6. S1P Intracellular Targets

The majority of S1P’s actions are exerted through its binding to the specific S1PRs present at the plasma membrane, but specific intracellular targets of relevance to cancer have been discovered. One of these is TRAF2, a component of the TNF-α/NF-κB signaling pathway. TRAF2 is an E3 ubiquitin ligase, and it has been shown that binding with S1P stimulates its ubiquitin ligase activity [134]. SphK1 is also necessary for the ubiquitination of RIP1 and the following activation of NF-κB induced by TNF-α. Xiong et al. suggested a different mechanism for the activation of TRAF2 activity that demonstrated that, in macrophages derived from bone marrow, SphKs are not necessary for the activation of NF-κB induced by TNF-α [135]. In addition, Hait et al. demonstrated that S1P derived from SphK2 is able to bind and inhibit deacetylases 1 and 2, increasing histone acetylation [42]. These data are supported by a study performed in *Drosophila*, which have no S1PRs, demonstrating that high levels of nuclear S1P cause a decrease in histone deacetylase activity and increase histone acetylation [136].

## 5. Conclusions

Multiple factors drive, perpetuate, and exacerbate the pathogenesis of cancer, including inherited predisposition and environmental interactions. Bioactive lipids, with their associated metabolism and regulatory pathways, have emerged as important players in the pathogenesis and potential treatment of cancer [4,5,6,8,9]. Specifically, S1P regulates a myriad of processes crucial for cancer progression such as proliferation, angiogenesis, metastasis, survival and drug resistance [7,8,12,137,138,139,140,141,142,143,144,145,146]. The modulation of sphingolipid metabolism, particularly S1P, can provide a useful strategy for treating cancer. S1P levels are significantly increased in many tumors; Spiegel and her collaborators highlighted the crucial role of the sphingolipid (S1P/Cer) rheostat in the regulation of cell fate. In particular, the balance between S1P and Cer levels is crucial in the initiation, progression, and drug sensitivity of cancer [6,10]. It has been demonstrated that decreased production of S1P is fundamental in cancer biology [13,15,29,30,31,32]. Based on the above, the targeting of enzymes involved in S1P metabolism is critical to control cancer growth. In particular, it has been shown that the oncogenic role of S1P is associated with the regulation of SphK1. SphK1 has been shown to regulate tumorigenic properties and cancer progression by modulating apoptosis, autophagy, proliferation, migration, invasion, angiogenesis, and inflammation. Moreover, Van Broocklin demonstrated that a high expression level of SphK1 correlates with poor survival in patients affected by glioblastoma multiforme [50].

Altogether, these findings indicate that SphK1 is a promising target for cancer therapy [70,86]. In contrast to SphK1, SphK2’s role in disease, and in particular in cancer, is not fully understood [6,73]. In addition, the levels of S1P lyase are downregulated in different human cancers and there is an inverse correlation with clinical outcomes and resistance to treatment [6,93]. Furthermore, the expression of S1P phosphatases is decreased in many types of cancer [6]. In particular, increased S1P levels in tumors were found to be directly correlated with high levels of SphK1 and low levels of SPP2, further suggesting complex coordination in the regulation of sphingolipid metabolism [97].

The inhibition of the S1PRs/S1P transporters (SpnS2 and/or ABC) could be another strategy to modulate S1P signaling. Sphingolipids and the enzymes related to their metabolism are involved in the biogenesis and release of extracellular vesicles that may play a role in the transport and release of pro-tumoral molecules [147,148,149,150,151]. It has recently been recognized that exosome-based therapy might be useful in cancer therapy [152]. The findings summarized highlight the fact that we need to continue to study the specific mechanisms of sphingolipids involved in the regulation of cancer to find therapies based on sphingolipid metabolism. Recently, Rixe et al. performed a human phase I study with the sphingolipid metabolism regulator BXQ-350, a nanovesicle formulation of saposin C; it increased Cer levels, decreased S1P levels, and demonstrated prolonged progression-free survival in a patient with gliomas with good tolerance [153].

One of the challenges in developing sphingolipid-based cancer therapies is the selective targeting of cancer cells, since sphingolipid metabolism is critical in all cells and might behave differently in different types of tumors, thus impacting the success of therapy targeting these pathways. Moreover, it should be considered that the modulation of sphingolipid metabolism may have side effects due to its involvement in other fundamental physiological processes. Overall, targeting the S1P metabolic pathway in cancer is a cutting-edge area of research, as supported by clinical trials exploring its potential as a therapeutic strategy. However, much remains to be understood before widespread clinical application. In summary, all this knowledge highlights the fact that further research is needed to better understand the complex interactions between sphingolipid metabolism and cancer progression, in order for us to design more effective and safe therapeutic strategies.

## Figures and Tables

**Table 1:** List of clinical trials targeting sphingosine 1-phosphate (S1P) pathways in different types of cancer.

Drug/Target	Cancer Type	Clinical Trial Phase	Trial Focus	Trial ID
Safingol (SK1-I)/SK1 inhibitor	Various advanced solid cancers	Phase I completed	Determine the maximum tolerated dose when administered cisplatin	NCT00084812
	Various solid cancers relapsed malignancies	Phase I terminated	Determine the maximum tolerated dose in combination with fenretinide	NCT01553071
ABC294640 (Opaganib/SK2 inhibitor	Cholangiocarcinoma	Phase II completed	Determine the response rate and the disease control rate	NCT03377179
	Multiple myeloma	Phase I/II completed	Assess the drug’s safety and identify the maximum tolerated dose	NCT02757326
	Various solid cancers; hepatocellular carcinoma	Phase II completed	Assess safety and determine the maximum tolerated dose and dose-limiting toxicities	NCT01488513
	Prostate cancer	Phase II completed	Investigational study in patients who have experienced disease progression while receiving abiraterone or enzalutamide	NCT04207255
Fingolimod (FTY720)/non-selective agonist of S1P receptors, with the exception of S1P2, selective functional antagonist of S1P1	Non-small-cell lung cancer	Phase II Not yet recruiting	Investigating effects with chemo-immunotherapy agents	NCT06424067
	Glioblastoma	Phase I completed	Investigation as part of combination therapy with radiation and temozolomide	NCT02490930
Mocravimod (KRP203)/S1P1 and S1P5 receptor agonist	Hematological malignancies	Phase I completed	Evaluating safety, tolerability, and pharmacokinetics in allogeneic hematopoietic stem cell transplantation recipients	NCT01830010
Sonepcizumab/anti-S1P monoclonal antibody	Various solid tumors	Phase I completed	Evaluating safety, tolerability, and the highest dose that can safely be administered to patients with cancer who are no longer being helped by standard treatments	NCT00661414
	Renal carcinoma	Phase II terminated	Evaluating efficacy, safety, and tolerability	NCT01762033
